# Robust electrocardiogram delineation model for automatic morphological abnormality interpretation

**DOI:** 10.1038/s41598-023-40965-1

**Published:** 2023-08-23

**Authors:** Siti Nurmaini, Annisa Darmawahyuni, Muhammad Naufal Rachmatullah, Firdaus Firdaus, Ade Iriani Sapitri, Bambang Tutuko, Alexander Edo Tondas, Muhammad Hafizh Permana Putra, Anggun Islami

**Affiliations:** 1 Intelligent System Research Group, Faculty of Computer Science, Universitas Sriwijaya, Palembang, 30139 Indonesia; 2 Department of Cardiology and Vascular Medicine, Dr. Mohammad Hoesin Hospital, Palembang, 30126 Indonesia

**Keywords:** Medical research, Machine learning

## Abstract

Knowledge of electrocardiogram (ECG) wave signals is one of the essential steps in diagnosing heart abnormalities. Considerable performance with respect to obtaining the critical point of a signal waveform (P-QRS-T) through ECG delineation has been achieved in many studies. However, several deficiencies remain regarding previous methods, including the effects of noise interference on the performance degradation of delineation and the role of medical knowledge in reaching a delineation decision. To address these challenges, this paper proposes a robust delineation model based on a convolutional recurrent network with grid search optimization, aiming to classify the precise P-QRS-T waves. In order to make a delineation decision, the results from the ECG waveform classification model are utilized to interpret morphological abnormalities, based on medical knowledge. We generated 36 models, and the model with the best results achieved 99.97% accuracy, 99.92% sensitivity, and 99.93% precision for ECG waveform classification (P-wave, QRS-complex, T-wave, and isoelectric line class). To ensure the model robustness, we evaluated delineation model performance on seven different types of ECG datasets, namely the Lobachevsky University Electrocardiography Database (LUDB), QT Database (QTDB), the PhysioNet/Computing in Cardiology Challenge 2017, China Physiological Signal Challenge 2018, ECG Arrhythmia of Chapman University, MIT-BIH Arrhythmia Database and General Mohammad Hossein Hospital (Indonesia) databases. To detect the patterns of ECG morphological abnormalities through proposed delineation model, we focus on investigating arrhythmias. This process is based on two inputs examination: the P-wave and the regular/irregular rhythm of the RR interval. As the results, the proposed method has considerable capability to interpret the delineation result in cases with artifact noise, baseline drift and abnormal morphologies for delivering robust ECG delineation.

## Introduction

Electrocardiography (ECG) signals are a primary criterion for medical practitioners to provide information such as rhythm and heart rate^1^. The development of a reliable, accurate, noninvasive and robust method for automatic ECG signal delineation could assist cardiologists in the study of patients with heart disease. A normal waveform of an ECG signal consists of P-waves, QRS complexes, and T-waves^2^. To diagnose some heart abnormalities, physicians commonly observe ECG morphology manually. Recognizing abnormal ECG signals visually is arduous because of their varying morphology, and ECG signals are incredibly susceptible to noise^3^. ECG signals are very weak bioelectric signals. There are three main types of noise^4,5^, electrode motion artifacts, muscle artifacts, and baseline drift, that commonly accompany ECG signals. This noise may influence the shape characteristics of the amplitude and baseline of ECG signals, increasing the difficulty of delineating ECG signals.

An ECG signal is a type of time series data that changes periodically over time, and the aim of ECG delineation is to find key feature points^6^. ECG delineation is a crucial step in processing ECG signals and helps to identify the critical points that indicate the interval and amplitude locations in each wave morphology^6^. There are two main ECG delineation methods: digital signal processing methods^7–9^ and intelligent processing methods^3,10–19^. Many researchers have performed considerable research in both types of ECG delineation^3,7–19^. However, several deficiencies remain regarding the delineation of ECG signal methods: (1) some existing methods require prespecified thresholds or other assumptions^8^, (2) a limited number of studies have investigated the role of medical knowledge in delineation models as a decision^20^, and (3) the effects of noise have not been closely examined^18^. Hence, an automatic and simple ECG signal delineation method with robust performance is desirable.

This study proposes intelligent signal ECG processing for a robust delineation method. ECG delineation consists in computing the onset and offset locations for each ECG wave (P-QRS-T waves). By this approach, classifying P-QRS-T waves is still being worked on by clinical practice^6,10^. We add the role of medical knowledge to provide an interpretable delineation result that automatically identify abnormal morphologies, with a focus on arrhythmias. Medical knowledge is essential in the interpretation of ECG morphological abnormalities. It plays a crucial rule in accurately interpreting and analyzing ECG signals by providing an understanding of the heart's electrical activity and its physiological and pathological aspects. This knowledge allows for the identification and categorization of different ECG waveforms, such as P waves, QRS complexes, and T waves, which provide valuable information about electrical conduction and potential cardiac abnormalities. The main contributions of this paper are as follows:

- Proposing a robust ECG delineation model based on a convolutional recurrent network to highly precisely classifying P-waves, QRS-complexes, and T-waves;
- Interpreting ECG morphological abnormalities, with a focus on arrhythmias, using the proposed delineation model based on the P-wave and the regular/irregular rhythm of the RR interval as rules guided by medical knowledge; and
- Implementing a grid search optimization algorithm to increase the delineation model performance.

## Materials and methods

This section provides a description of the study design which is related to two main tasks;

(i) generate a robust delineation model based on a convolutional recurrent network with grid search optimization, aiming to classify the precise P-QRS-T waves through beat-to-beat.
(ii) interpret the ECG delineation results, using rules of medical knowledge to make a decision.

**Figure 1:**
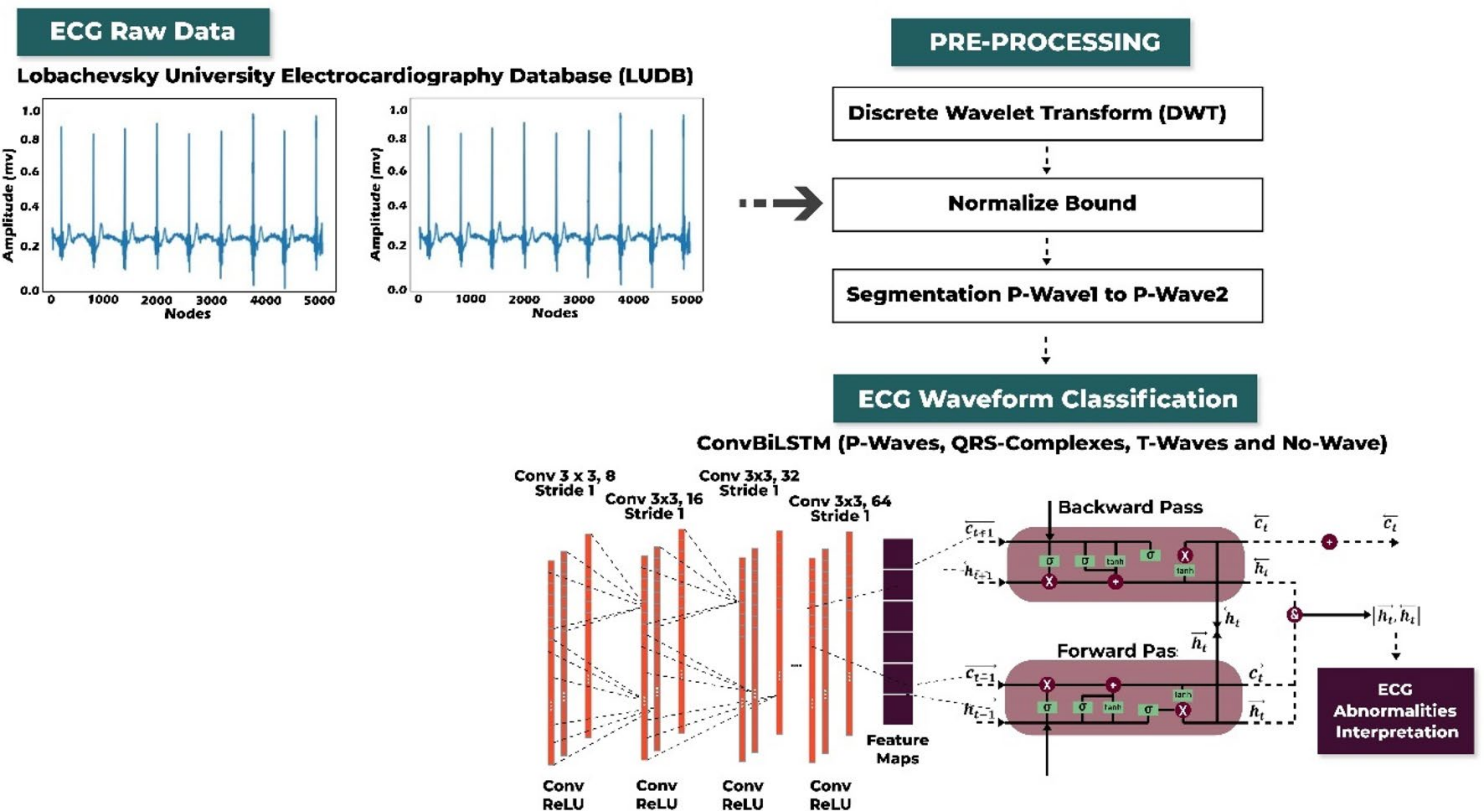
The research methodology of ConvBiLSTM.

The research methodology is described in detail to offer a clear understanding of the experimental procedures, as illustrated in Fig. 1. This methodology workflow consists of: (i) generate the ECG delineation model using an experimental dataset from the Lobachevsky University Electrocardiography Database (LUDB) that has been preprocessed by noise cancelation with discrete wavelet transform (DWT). In addition, normalized bounds is applied for amplitude range normalization and segmentation to obtain the current beat to the next beat (beat-to-beat), e.g., from onset of P-wave1 to onset of P-wave2, onset of P-wave2 to onset of P-wave3 and so on; (ii) propose Convolution Bidirectional Long Short-Term Memory (ConvBiLSTM) model with hyperparameter tuning optimization to classify P-waves, QRS-complexes, T-waves, and isoelectric line (no waves) from beat-to-beat; and; (iii) interpret ECG morphological abnormalities, with a focus on arrhythmias, through delineation approach based on two inputs examination: the P-wave and the regular/irregular rhythm of the RR interval.

### ECG signal dataset

In this study, there are three categorical of experimented datasets:

1. To generate the ECG delineation model, we implemented LUDB. LUDB is an ECG signal database that consists of 200 10-s 12-lead (I, II, III, V1, V2, V3, V4, V5, V6, aVR, aVL,and aVF) records^21^. In LUDB, the boundaries and peaks of P-waves, QRS complexes, and T-waves were marked and annotated by two verified cardiologists to represent the distinct morphologies of an ECG signal. Each record has a length of 10 s and was digitized at 500 Hz. Of the 58,429 total waveforms, there were 16,797 P-waves, 21,966 QRS complexes and 19,666 T-waves. We classified the three main ECG waveforms and isoelectric line (no wave). For data splitting, the model was generated by splitting 90% of the data for training and the remaining data for validation.
2. To test our delineation model, we have explored QT Database (QTDB) as testing set (unseen) to provide an unbiased evaluation of a best model fit on the training dataset^22^. QTDB has commonly experimented for ECG delineation task in several researches^6,10,18,19^. QTDB has been used due to it was provided the beats that manually annotated by cardiologists. The annotation of the onset, peak and offset of the P-wave, the onset and offset of the QRS-complex, the peak and offset of the T-wave, and (if present) the peak and offset of the U-wave have available.
3. To evaluate the proposed ECG delineation model, we attempt to interpret the normal sinus rhythm (NSR), atrial fibrillation (AF) and atrial flutter (AFL) from five ECG databases, including LUDB, the PhysioNet/Computing in Cardiology Challenge 2017^23^, China Physiological Signal Challenge 2018^24^, ECG Arrhythmia of Chapman University^25,26^ and General Mohammad Hossein Hospital (Indonesia) databases. Aforementioned records have been annotated by experts as NSR, AF and AFL, respectively. NSR is rhythm of the healthy human heart. The atria beat irregularly in AF, while AFL, it may experience four atrial beats for every ventricular beat because the atria beat regularly but faster than usual and more frequently. We have also utilized other ECG morphological abnormalities that related to irregular heart rate; i.e., sinus bradycardia (SBR), supraventricular tachyarrhythmia (SVTA), ventricular trigeminy (T), and ventricular tachycardia (VT), from the records of the MIT-BIH Arrhythmia Database^27^ (Table 1). SBR, SVTA, T, and VT are arrhythmias that are related to irregular heartbeats, in which SB is slower than expected, and SVTA and VT are much faster than normal. T is an abnormal heart rhythm that occurs when every third beat is a premature ventricular contraction.

**Table 1:** The experimented ECG records for abnormality morphology interpretation.

Database	ECG Records			Information
	Rhythm	Total records	Subject records	
LUDB^21^	NSR	142	2–5, 7, 9–11, 13–21, 23, 25–26, 28–29, 31–34, 36–37, 40, 42–43, 45–46, 48–50, 53, 55–58, 60–62, 64–69, 72–81, 84–87, 89–92, 94, 97–98, 100, 102, 104–107, 111, 113, 116, 118–124, 126, 130–131, 135–139, 141–145, 147–155, 157, 159, 161–168, 171, 174–177, 179–183, 185–187, 190–196, 198–200	Training and validation set to generate the ECG waveform classification (delineation). For data splitting, the model was generated by splitting 90% of the data for training and the remaining data for validation
	AF	14	8, 38, 44, 51, 83, 88, 93, 95, 96, 101, 110, 112, 129, and 173	
	AFL	3	35, 52 and 103	
QTDB	NSR	10	1–10	To provide an unbiased evaluation of a best model as testing set (unseen)
PhysioNet/Computing in Cardiology Challenge 2017^23^	AF	14	1, 2, 3, 4, 5, 6, 7, 8, 9, 10, 11, 12, 13, 14	To interpret the morphological abnormality from ECG delineation results based on medical knowledge
China Physiological Signal Challenge 2018^24^	AF	17	1, 2, 3, 4, 5, 6, 7, 8, 9, 10, 11, 12, 13, 14, 15, 16, 17	
ECG Arrhythmia of Chapman University^25,26^	AFL	3	1, 2, 3	
MIT-BIH Arrhythmia Database^27^	SBR	1	232	
	SVTA	1	207	
	T	1	119	
	VT	1	200	
Central General Hospital Dr. Mohammad Hoesin, Indonesia	AF	2	1, 2	

The research methodology of this study can be explained as follows:

### ECG preprocessing

The measurement and analysis of the ECG signal are challenging due to noise. Noise can be generated from various sources, such as motion and muscle artifacts, powerline interference, and baseline drift. We implemented the DWT to address the ECG noise cancelation problem. The quality of the denoising process depends on the wavelet function, decomposition level, threshold selection and reconstruction^4^. In this study, we compared some wavelet functions, such as *sym5, sym6, sym7, sym8, db2, db4, db5, db6, db7, bior1.3, bior6.8, bior3.5, haar* and *coif5* (Table 2). To obtain the selected wavelet function, we calculated the signal-to-noise ratio (SNR) to compare the level of an output signal (desired signal) to the level of background noise^28^. The denoising efficiency is measured using the SNR. SNR provides information about the signal quality. Among the experimental wavelet functions, the highest output SNR value was that of *coif5*, with 8.56 decibels (dB). The input SNR is defined as:

1 $$ SNR_{i} = 10\log_{10} \left[ {\frac{{\sum\nolimits_{n} {x^{2} } (n)}}{{\sum\nolimits_{n} {r^{2} (n)} }}} \right] $$

**Table 2:** The SNR results of wavelet functions.

Wavelet functions	Averaged SNR (dB)
*sym5*	8.29200
*sym6*	8.47519
*sym7*	8.43995
*sym8*	8.50277
*db2*	8.19673
*db4*	8.44843
*db5*	8.26938
*db6*	8.24373
*db7*	8.41857
*bior1.3*	8.44209
*bior6.8*	8.41857
*Haar*	8.41857
*bior3.5*	8.44209
*coif5*	8.56691

The output SNR $(SNR_{o} )$ is given by the following equation:

2 $$ SNR_{o} = 10\log_{10} \left[ {\frac{{\sum\nolimits_{n} {x_{d}^{2} (n)} }}{{\sum\nolimits_{n} {(x_{d} (n) - x(n))^{2} } }}} \right] $$

where $x(n)$ is the original with length $n$, $r(n)$ is the added noise signal, and $x_{d} n$ is the denoised signal.

For decomposition level, we have used 8 levels of decomposition, in which the largest frequency sequence ranges from level 1 to level 8. In this study, the denoising was developed following the soft thresholding. It is first sets to zero the elements whose absolute values are lower than the threshold, and then shrinks the nonzero coefficients toward 0.

After ECG noise cancellation, we normalized the amplitude range for efficient computation. We have applied normalize bound, one of the processing subpackage contains signal-processing tools (waveform-database, WFDB) for reading, writing, and processing WFDB signals and annotations. In this method, the value of the signal data with the lower limit (zero) and upper limit (one) values was changed.

**Figure 2:**
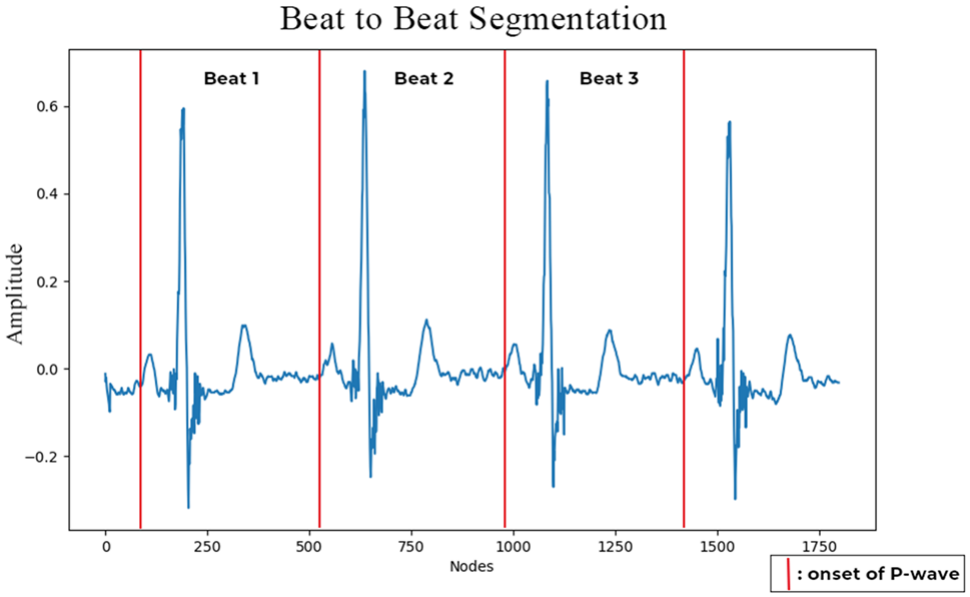
The beat-to-beat segmentation process.

In the last preprocessing step, we segmented the ECG records beat-to-beat (from the onset of P-wave 1 to the onset of P-wave2, onset of P-wave2 to onset of P-wave3 and so on) (refer to Fig. 2). We assume one beat consists of at least one R-peak. The segmentation process is referred by expert annotation. The input shape of each beat was set to 512 nodes.

### ConvBiLSTM model

The primary purpose of ECG signal delineation is to classify P-QRS-T waves. The higher amplitude of the QRS complex is usually simple to identify. It differs from that of P- and T-wave delineation, which is particularly challenging due to its lower amplitude and occasionally noise-accompanied nature. In this study, we were concerned with obtaining the precise locations of P-waves and RR-intervals because it is essential for ECG morphological abnormality such as arrhythmias.

In this study, we experimented with ConvBiLSTM as the ECG delineation architecture^18^. This architecture consisted of four convolution layers for feature extraction and BiLSTM as the ECG waveform classifier. For the LSTM input layer must be three dimensions. The meaning of the three input dimensions are; samples, timesteps and features. The total number of nodes in one beat was 512 (as features), with a timestep of (250, 1). If the one segmented beat had fewer than 512 nodes, we added To validate our proposed model, we also tested the other AF database using unseen records (records that were not used for training and validation a zero value for the remaining values (zero-padding technique). The timesteps of input with dimension (250, 1) were fed into the convolution layer. The input of ConvBiLSTM is the ECG waveform which bounded by a vector label indicating the class of each node. The class label is formed in vector with size of (250, 1). We adjusted ConvBiLSTM for P-wave, QRS complex, T-wave, and no wave classification. The ConvBiLSTM was fed into the convolution layer equipped with rectified linear unit (ReLU) and softmax activation functions for the hidden and output layers, respectively. The Adam optimizer is used for optimization techniques in deep learning for stochastic gradient descent, and categorical cross-entropy is used as a loss function.

The one-dimensional forward propagation of convolutional neural network (CNN) can be expressed as follows^29^:

3 $$ x_{k}^{l} = b_{k}^{l} = \sum\limits_{i - 1}^{{N_{l - 1} }} {conv} \quad 1d(w_{ik}^{l - 1} ,s_{i}^{l - 1} ) $$

where $x_{k}^{l}$ is the input, $b_{k}^{l}$ is the bias of the *k*th neuron at layer *l*, $s_{i}^{l - 1}$ is the output of the *i*th neuron at layer *l* − 1, and $w_{ik}^{l - 1}$ is the kernel from the *i*th neuron at layer *l* − 1 to the *k*th neuron at layer *l*.

The BiLSTM can be expressed as follows:

4 $$ h_{t}^{f} = \tan h(W_{xh}^{f} xt + W_{hh}^{f} h_{t - 1}^{f} + b_{h}^{f} ) $$

5 $$ h_{t}^{b} = \tan h(W_{xh}^{b} xt + W_{hh}^{b} h_{t - 1}^{b} + b_{h}^{b} ) $$

6 $$ y_{t} = W_{hy}^{f} h_{t}^{f} + W_{hy}^{b} h_{t}^{b} + b_{y} $$

where to generate the output *y*_*t*_, the forward hidden layer $h_{t}^{f}$ and the backward hidden layer $h_{t}^{b}$ are combined.

### ECG morphological abnormality interpretation

In this study, we interpreted ECG morphological abnormalities, with a focus on arrhythmias, using the ECG delineation approach. Currently, AF and AFL are the two arrhythmias that occur most often. Since AF and AFL conditions have similar physiological characteristics and are frequently present in more than half of AFL patients, which both are rapid upper chamber arrhythmias, they are also frequently linked to other cardiovascular disorders, including stroke and myocardial infarction^30^. AF and AFL are sharing similar physiological characteristics is because they both involve abnormal electrical activity in the atria of the heart. In both conditions, the normal coordinated rhythm of the atria is disrupted, leading to irregular or rapid heartbeats. This similarity in the underlying electrical abnormalities can result in overlapping symptoms and diagnostic features. A common AF and AFL diagnostic method uses a visual evaluation of ECG. Other types of arrhythmias are SBR, SVTA, T, and VT, which they were related to irregular heart rate.

Therefore, based on the proposed delineation model, we examine two inputs: the P-wave and the regular/irregular rhythm of the RR interval. We can identify the P-wave pattern from the delineation result, but the QRS complex must be analyzed to determine any irregular heart rate. Adults typically have a resting heart rate (HR) between 60 and 100 beats per minute (BPM). A resting HR of less than 60 BPM is referred to as bradycardia (slow ventricular response), and that consistently above 100 BPM reflects a rapid ventricular response (tachycardia)^31,32^. In our previous work^20^, we stated that the regular rhythm has a pattern (normal, slow, or rapid ventricular response), and an irregular rhythm indicates no pattern on the ECG signal. Therefore, the NSR, AF, AFL and other arrhythmia were interpreted according to the medical knowledge rules^33,34^:

(i) if the P-wave was present and the rhythm was regular, then the condition was the NSR;
(ii) if the P-wave was absent and the rhythm was irregular, then the condition was the AF;
(iii) if the P-wave was absent and the rhythm was regular, then the condition was the AFL; and
(iv) if the P-QRS-T wave was present and the rhythm was irregular, then the condition was others arrhythmia.

### Evaluation metrics

In this study, we have measured the ECG waveform classification (P-QRS-T waves) based on evaluation metrics for supervised learning, i.e., accuracy (Acc), sensitivity (Sen) and precision (Pre). Using different metrics for performance evaluation, we able to improve the proposed ConvBiLSTM model’s overall predictive power before we test on unseen set. In this study, we have calculated Acc, Sen and Pre for training, validation and unseen sets. Acc defines as the ratio of the number of true predictions and the total number of predictions. Sen defines how many of actual positive class, we able to predict correctly with the proposed model. Pre explains how many of the correctly predicted class actually turned out to be positive. We can define the Acc, Sen and Pre based on mathematical functions below:

7 $$ Acc = \frac{TP + TN}{{TP + TN + FP + FN}} $$

8 $$ Sen = \frac{TP}{{TP + FN}} $$

9 $$ \Pr e = \frac{TP}{{TP + FP}} $$

where TP is the True Positive, TN is the True Negative, FP is the False Positive, FN is the False Negative.

## Results

To comprehensively explain the results obtained from our experiment, we divided them into two main discussions; (i) delineation model performance, and (ii) interpretation of ECG delineation.

### Delineation model performance

Using the 200 records in LUDB and ConvBiLSTM, an ECG delineation model was generated. For the ground truth of LUDB, we segmented the ECG signal to beat-to-beat; it is the process to segment current beat to the next beat (beat-to-beat), from onset of P-wave1 to onset of P-wave2, onset of P-wave2 to onset of P-wave3 and so on (Fig. 2). The total number of nodes in one beat was 512, with a timestep of (250, 1). If the one segmented beat had fewer than 512 nodes, we added a zero value for the remaining values (zero-padding technique). LUDB includes 12-lead ECGs. However, in this study, we only used single-lead ECGs, i.e., lead II. In some situations, lead II is usually the best lead in which to observe P-waves and is mostly used for arrhythmia interpretation.

In the design phase of the ConvBiLSTM model, the grid search optimization (GSO) algorithm was implemented for optimum hyperparameter selection. The GSO algorithm is a common approach to determine the best combination of hyperparameter values for DL models. The impact of each parameter combination on the performance of the model was computationally evaluated. To finetune the ConvBiLSTM architecture, the GSO algorithm was determined by the hyperparameter ranges specified in Table 3. As seen in Table 3, 36 models were tested based on the batch size (8, 16, and 32), learning rate (10^–3^, 10^–4^, and 10^–5^) and epochs (100, 200, 300, and 400) parameters.

**Table 3:** The GSO algorithm combination of hyperparameters and value ranges.

	Hyperparameters to optimize	Values ranges
1	Number of batch size	(8, 16 and 32)
2	Number of learning rate	(10^–3^, 10^–4^, and 10^–5^)
3	Number of epochs	(100, 200, 300 and 400)

**Figure 3:**
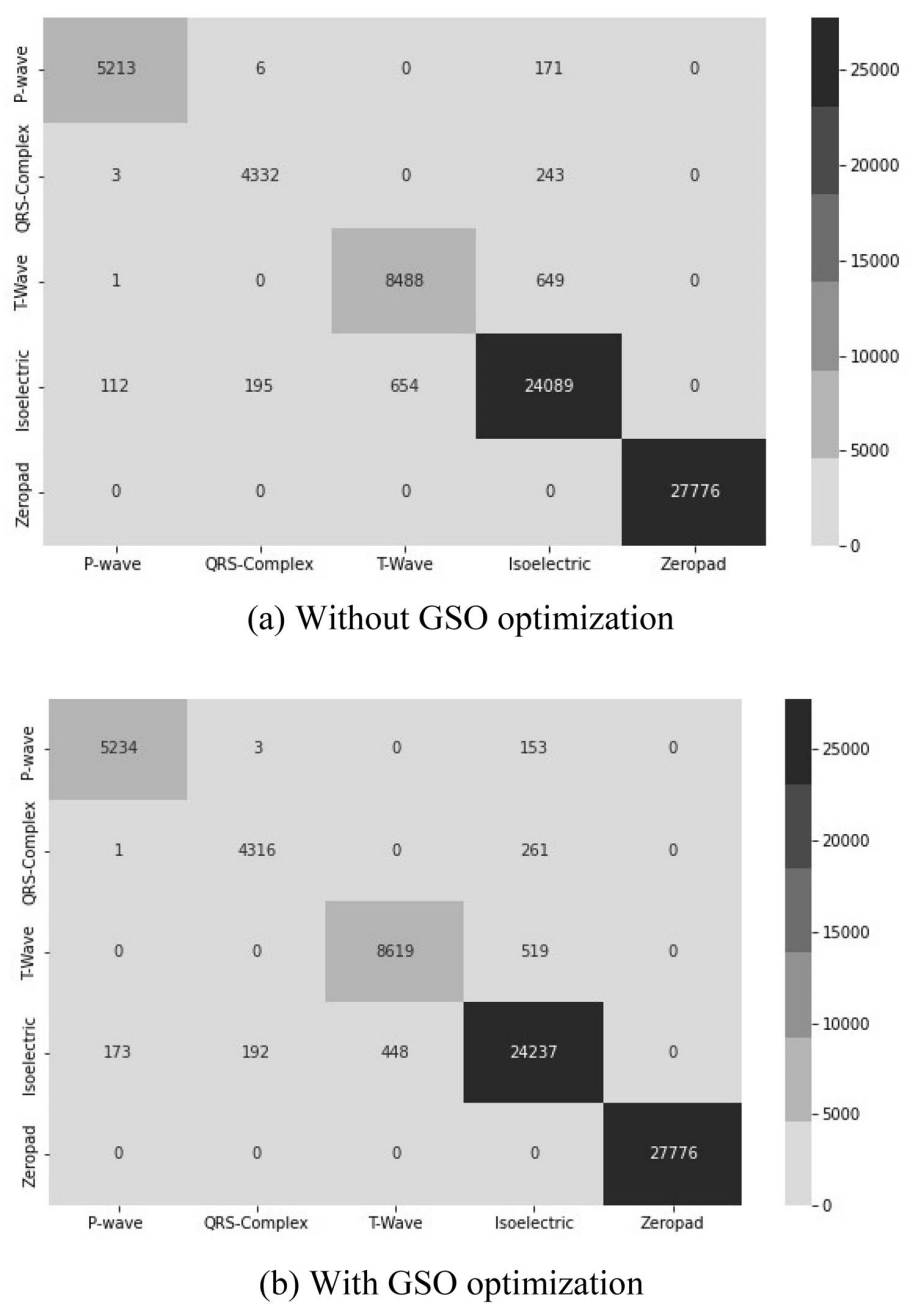
Confusion matrix of ConvBiLSTM.

To validate our ConvBiLSTM with GSO, we experimented using ConvBiLSTM without GSO. The results of the confusion matrix (CM) are presented in Fig. 3. Both ConvBiLSTM models falsely classified the isoelectric line (no wave) as a P-wave, QRS-complex, T-wave and vice versa. However, the total number of misclassifications in the ConvBiLSTM model without GSO (Fig. 3a) was higher than that with GSO (Fig. 3b). Among all ECG waveforms, the misclassification of T-waves was dominant due to the maximal errors observed for the offset of T-waves.

**Figure 4:**
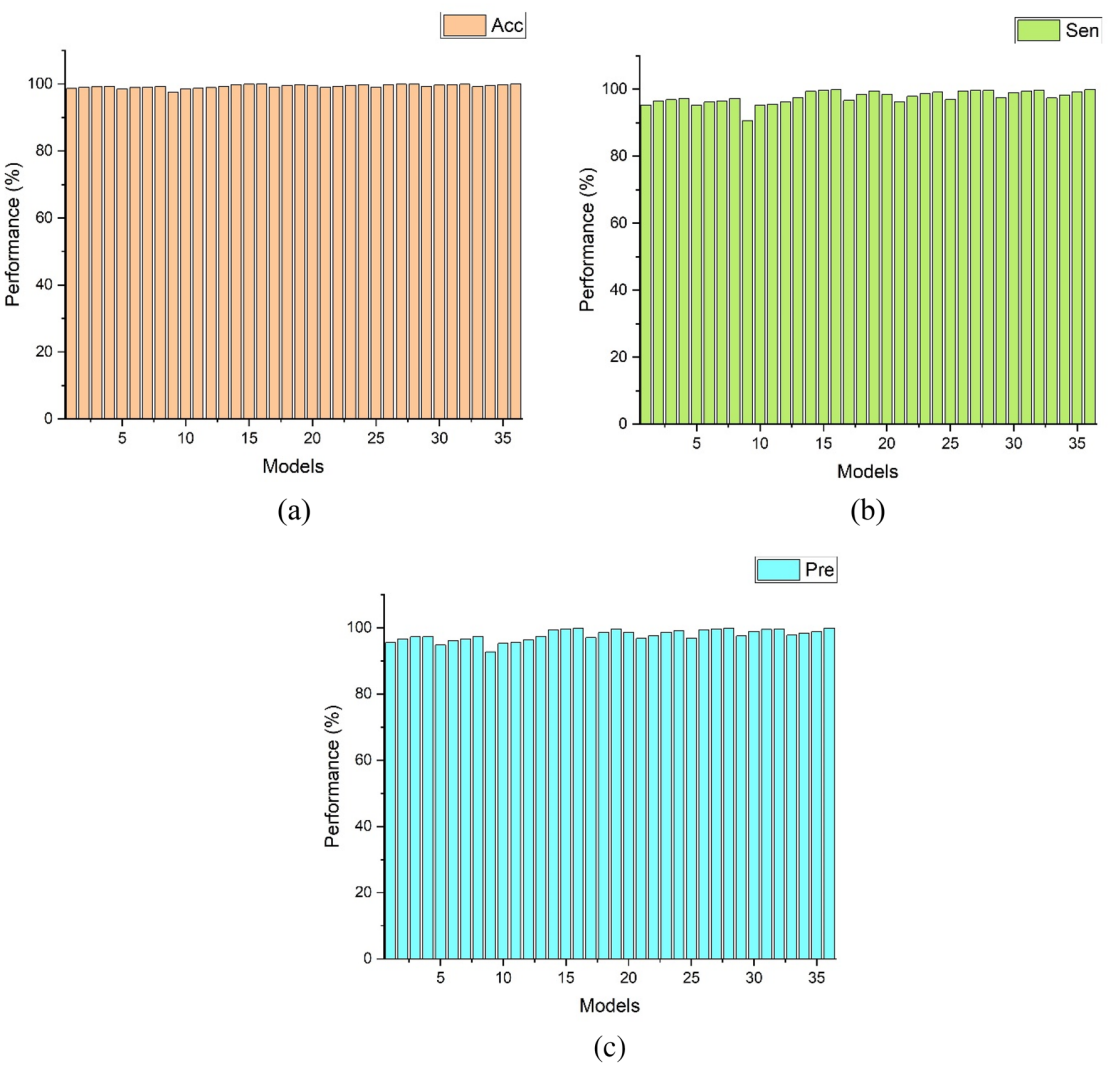
The performance results of 36 ConvBiLSTM models using the GSO algorithm from validation set.

The ConvBiLSTM with the GSO algorithm generated 36 models by combining the main hyperparameters (batch size, learning rate and epochs). The results of 36 models generated using ConvBiLSTM with the GSO algorithm from validation set are presented in Fig. 4. Figure 4 shows three metric evaluations (Acc (red), Sen (green), and Pre (blue)), with ranges above 90%, and the highest value was close to 100%. Among the 36 models based on hyperparameter tuning, the last model (Model 36) was proposed for NSR, AF, and AFL interpretation. The hyperparameters of the best model were constructed using a batch size of 32, a learning rate of 10^–3^, and 400 epochs. All the results using Model 36 achieved above 99.92% for Acc, Sen, and Pre.

**Figure 5:**
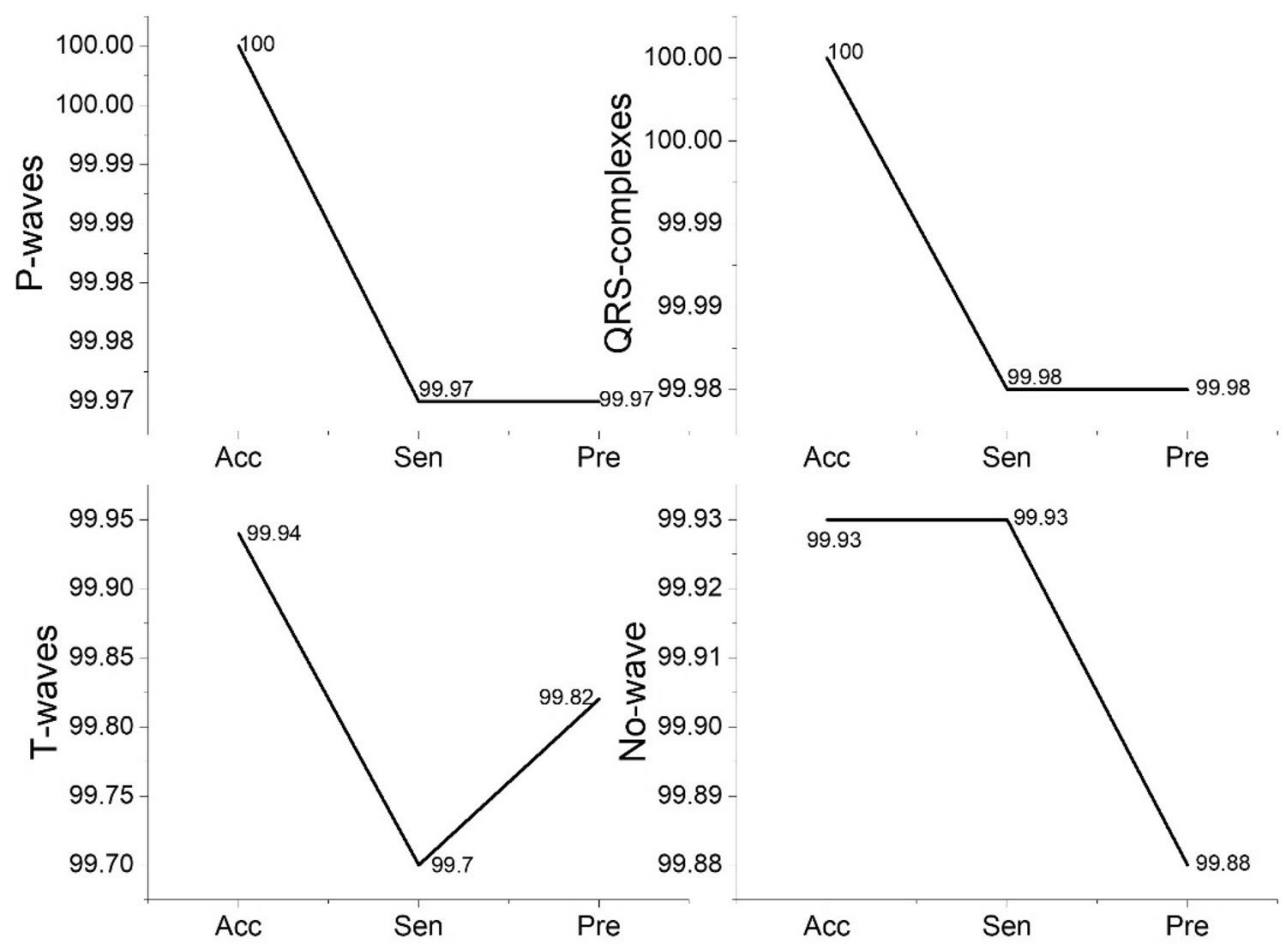
The Acc, Sen and Pre of the ECG waveform class of the proposed model.

The distribution of each class performance results in the best model of ConvBiLSTM with the GSO algorithm using validation set (Model 36) is shown in Fig. 5 and Table 4. The performance results for P-waves, QRS-complexes, T-waves and no waves are excellent. We successfully classified the P-waves and QRS complexes with 100% Acc (Table 4). We are concerned with P-wave and RR-interval examinations. However, the results show that the model can be used to interpret arrhythmias. The interpretation of QRS complexes was affected by examining the RR interval. For the T-wave and no wave classes, the results are still good. T-waves represent the ventricular myocardium repolarization that is used to diagnose pathology ventricular arrhythmias.

**Table 4:** Each class performance results in the best model of ConvBiLSTM with the GSO algorithm using validation set.

Metrics	Performance results (%)					
	P-wave	QRS-complex	T-wave	Isoelectric line	Zero-padding	Average
Acc	100	100	99.94	99.93	100	99.97
Sen	99.97	99.98	99.7	99.93	100	99.92
Pre	99.97	99.98	99.82	99.88	100	99.93

In our previous research^18,19^, the average Sen was only 98.91%^18^ for P-waves, QRS-complexes, T-waves, and no waves. Additionally, in^19^, we updated ConvBiLSTM using the unsupervised denoising algorithm denoising autoencoder (DAE), and the results were approximately 98.59% for Acc, Sen, and Pre. The performance results in this study successfully outperformed those of our previous studies^18,19^. Due to the outstanding results, the proposed model was used to interpret ECG morphologies, such as the NSR, AF and AFL.

To validate and provide an unbiased evaluation of our robust delineation model, we have tested the ConvBiLSTM to testing set (unseen) by using QTDB. To delineate ECG waveform, a complete P-QRS-T wave must be required to identify critical points, which they were marked by interval and amplitude locations of each wave morphology. The records of NSR mostly provide a normal heartbeat that produces a regular, identifiable pattern: P-QRS-T waves. Therefore, in this study, we limited only tested 10 records of NSR (*sel16265, sel16272, sel16273, sel16420, sel16483, sel16539, sel16773, sel16786, sel16795, sel17453*), which it contains the automatic waveform onsets and offset in signal, within a normal range of regular pattern.

The total beats of NSR records in QTDB are 300, and all records were sampled at 250 Hz. Each class performance results can be listed in Table 5. Table 5 shows the performance results in several class are decreased. Compared to performance results using validation set (Table 4), with testing set, the average of Acc, Sen and Pre were decreased 1.73%, 7.29%, and 13.95%, respectively. The most significant occurs in T-wave, with the lowest result is only achieved 71.95% Pre. A normal T-wave overlapped with other T-wave characteristics, i.e., inverted T-wave, only upwards, only downwards, biphasic negative–positive, or biphasic positive–negative^2,35^. Aforementioned T-wave characteristics have maximal error, which it is observed for T-wave offset, whose delineation is a well-known hard problem^35^. Due to its problem, P-QRS wave shifted from onset and offset to the predicted results. Our proposed model tends to learn the regular T-wave positive features, which mostly appears in LUDB without considering other T-wave characteristics. Despite the aforementioned challenges, the proposed model consistently maintains a performance level above 85% Acc, Sen and Pre. This suggests that the model could be considered for application in clinical practice.

**Table 5:** Each class performance results in the best model of ConvBiLSTM with the GSO algorithm using testing set (QTDB).

Metrics	Performance results (%)					
	P-wave	QRS-complex	T-wave	Isoelectric line	Zero-padding	Average
Acc	99.43	98.84	97.16	95.74	100	98.24
Sen	92.59	98.47	90.65	81.36	100	92.63
Pre	88.66	73.65	71.95	95.65	100	85.98

### Interpretation of ECG delineation

The best ConvBiLSTM and optimization model was tested to interpret 142, 14, and 3 records for the NSR, AF and AFL, respectively. The interpretation of P-waves is arduous due to their low voltage amplitude and is often compared to noise. Therefore, the quality of P-wave interpretation must rely on restricting the temporal interval of interest from the QRS complex and T-wave. However, in this experimental work, the proposed model successfully achieved 100% truly interpreted NSR, AF, and AFL. The results are presented in Table 6. Table 6 shows that ConvBiLSTM had excellent interpretation for 142 LUDB records as NSR, 14 AF-infected records as AF and 3 AFL-infected records as AFL (only sample visualized). Table 6 presents the P-wave (blue), QRS complex (red), T-wave (yellow) and no wave (white) visualization. In the NSR records (records 2, 3 and 4), the presence of P-QRS-T waves and a regular pattern of rhythm was observed in the ECG signal. For the AF (records 51, 103, and 109) and AFL (records 35, 52, and 103) records, there is an absence of P-waves. Morphologically, both differences are the irregular atria beat in AF and the regular atria beat in AFL, but they are faster than usual and occur more often than those in the ventricles.

**Table 6:** The predicted results of NSR, AF, and AFL interpretation.

Records	LUDB	Delineation result	Delineation decision
2	NSR	NSR	Interpreted
3	NSR	NSR	Interpreted
4	NSR	NSR	Interpreted
51	AF	AF	Interpreted
93	AF	AF	Interpreted
109	AF	AF	Interpreted
35	AFL	AFL	Interpreted
52	AFL	AFL	Interpreted
103	AFL	AFL	Interpreted

To validate our proposed model, we also tested the other AF database using unseen records (records that were not used for training and validation) for morphological abnormality interpretation, i.e., the PhysioNet/Computing in Cardiology Challenge 2017 and the China Physiological Signal Challenge 2018 databases. There were 14 and 17 AF records for these databases, respectively. As a result, all 31 AF-infected records were successfully interpreted as AF. To present the results, we visualized the AF-interpreted sample in Table 7. The results cannot be affected by the lead and frequency sampling used. Although both databases have distinct lead and frequency sampling, our best model can interpet AF-infected AF. To interpret AFL, we tested the proposed model with three records from ECG Arrhythmia from Chapman University. With a 500 Hz sampling rate, the ECG records mostly consist of common arrhythmias and additional heart abnormalities. As a result, the proposed model can also interpet AFL-infected AFL.

**Table 7:** The sample predicted results of AF and AFL interpretation using the PhysioNet/Computing in Cardiology Challenge 2017, China Physiological Signal Challenge 2018, ECG Arrhythmia of Chapman University, MIT Arrhythmias Database and General Mohammad Hossein Hospital (Indonesia) databases.

ECG Database	Records	Delineation result	Delineation decision
The PhysioNet/Computing in Cardiology Challenge 2017	AF	AF	Interpreted
	AF	AF	Interpreted
	AF	AF	Interpreted
The China Physiological Signal Challenge 2018	AF	AF	Interpreted
	AF	AF	Interpreted
	AF	AF	Interpreted
ECG Arrhythmia of Chapman University	AFL	AFL	Interpreted
	AFL	AFL	Interpreted
	AFL	AFL	Interpreted
MIT Arrhythmias Database	SBR	SBR	Interpreted
	SVTA	SVTA	Interpreted
	T	T	Interpreted
	VT	VT	Interpreted
Central General Hospital Dr. Mohammad Hoesin, Indonesia	AF	AF	Interpreted
	AF	AF	Interpreted

In addition, we tested the MIT Arrhythmias Database to interpret arrhythmias (SBR, SVTA, T, and VT) using ConvBiLSTM. As visualized in Table 7, irregular heartbeats are present in the delineation result. The short/long RR-interval between beats is clearly observed, giving rise to irregular heart rhythms. Besides testing with public ECG databases, we also tested the proposed model with two ECG records from the General Mohammad Hossein Hospital (Indonesia) database. The ECG signals were recorded by a 12-Channel CardioCare 2000 Bionet ECG machine. All ECG records were digitized at 300 Hz. The morphologies of signals were fulfilled by noise and abnormal ECG patterns. Despite the challenging ECG records for examination, our proposed model can truly interpret AF.

## Discussion

In recent years, we generated an ECG delineation model by using digital signal processing methods (wavelet transform) and intelligent processing methods (DL). In^8^, we preliminarily experimented with the wavelet transform using DWT for feature extraction of the onset and offset of P-QRS-T waves. We used a conventional method to develop a low-complexity algorithm for ECG delineation. Based on feature analysis, we detected the onset and offset of P-QRS-T waves using a searching window technique based on the DWT threshold. We used eight levels of ECG reconstruction, where each level represents the P-QRS-T wave calculation. We detected the fiducial point of each wave using a searching window technique. However, the performance results are poor because they can be affected by feature analysis. A high degree of uncertainty and variability may exist due to the subjective aspect of the measurements in the segmentation and measurement phases.

To overcome this problem, we first used the DL algorithm for the ECG delineation task^18^. We generated the ConvBiLSTM algorithm for the delineation task to detect the onset and offset of P-waves, QRS-complexes, T-waves, and no waves^18^. The ConvBiLSTM algorithm combines the convolution layers of CNN for feature extraction and long short-term memory (LSTM) to classify ECG waveforms. The results reflect the excellent performance of the model; however, in a case of testing using an expert annotator, the precision of the ST-segment was 69.13%. Additionally, based on the limitations that we stated in^18^, ECG waveform classification was performed with and without considering specific heart abnormalities. Therefore, we improved ConvBiLSTM to detect the onset and offset of the main ECG waveforms considering heart abnormalities using morphology visualization. In^19^, the ConvBiLSTM algorithm was improved to detect heart abnormalities considering T-waves, i.e., T-wave alternans (TWAs). We experimented with a denoising autoencoder (DAE) as the noise cancelation method. As a result, we succeeded in detecting 20 of 30 records of synthesized ECGs with TWA^19^; unfortunately, there were still missed TWAs.

To improve our previous ConvBiLSTM algorithm, we generalized ConvBiLSTM using GSO to obtain a robust ECG delineation algorithm. The GSO algorithm was implemented to tune parameters to obtain optimal hyperparameters automatically. First, we generated 36 models by considering the batch size, learning rate and number of epochs. Second, among the resulting models, the best model (Model 36) was proposed based on its high-performance results in terms of Acc, Sen, and Pre, with performance results above 99.92%. Third, the best model was tested on 142, 14 and 3 records in LUDB for NSR, AF and AFL, respectively. As a result, the best model can interpret the records as NSR, AF and AFL. Fourth, the best model was also tested using other databases, i.e., PhysioNet/Computing in Cardiology Challenge 2017 and the China Physiological Signal Challenge 2018 databases. We were only concerned with interpreting AF in both databases. Additionally, a total of 31 AR records from both databases achieved 100% interpretation of AF. Although the LUDB, PhysioNet/Computing in Cardiology Challenge 2017 and the China Physiological Signal Challenge 2018 recordings were sampled at 500 Hz, 300 Hz, and 500 Hz, respectively, the differences in frequency sampling did not affect the performance of the best model (Model 36). In our previous work^18^, we stated that the limitation of our first-generation ConvBiLSTM needed to be adjusted to differentiate ECG frequency sampling and leads. With distinct frequency sampling and leads, in this study, we successfully improved the ConvBiLSTM performance results and interpreted NSR and arrhythmia (AF, AFL, SBR, SVTA, T, and VT) records with excellent results.

Generally, P- and T-wave detection in ECG signal recordings is difficult. P-wave detection is the most complicated part of delineation due to its high interpatient variability. However, in this study, we were concerned with ECG waveform classification to detect the onset and offset of P-QRS-T waves using a delineation approach. The best model learned the features from the labels (ground truth) in LUDB. We did not use a mathematical model to calculate the fiducial point of the ECG waveform. Additionally, conventional algorithms for QRS detection, such as Pan–Tompkins or wavelet transform, were not used. Instead, we proposed automatic delineation using DL to detect the onset and offset of ECG waveforms.

The varying records tested in this study contain some artifacts and interference. The PhysioNet/Computing in Cardiology Challenge 2017, China Physiological Signal Challenge 2018 and General Mohammad Hossein Hospital (Indonesia) databases, have abnormal artifacts that might distort morphological features, leading to a false diagnosis. Nevertheless, with the proposed methodology in this study, we were able to overcome this problem and achieve the true interpretation of normal and abnormal ECG morphologies to arrive at a decision.

The abovementioned problems of ECG can be improved to obtain a robust delineation model. In this study, we successfully developed a robust ConvBiLSTM that was tested using various ECG databases with different leads, frequency sampling, and types of noise. Our model is robust in interpreting ECG abnormalities, with focus on arrhythmias.

Some studies also experimented with DL for ECG delineation tasks with the same dataset. Excellent results above 97% Acc, Sen and Pre were obtained^17–19,36–38^. In comparison, for the performance of Acc, Sen and Pre, our ConvBiLSTM model outperformed the other DL techniques (Table 8). This study aimed to propose a robust ECG delineation model based on ConvBiLSTM to highly precisely classifying P-waves, QRS-complexes, and T-waves. Based on the results of delineation model, we have interpreted the ECG morphological abnormality. We generated 36 models, and the model with the best results achieved 99.97% accuracy, 99.92% sensitivity, and 99.93% precision for ECG waveform classification (P-wave, QRS-complex, T-wave, and isoelectric line class). We compared our results with those of other DL techniques and also improved our ECG delineation model compared with that in our previous works^8,18,19^. The improved ConvBiLSTM model was combined with the simplest optimization hyperparameter tuning model. A grid of hyperparameter values was set up, and for each combination, the model was trained and scored using validation data. In terms of improvement, for P-waves, the results of Acc, Sen and Pre increased from those in our previous works^18,19^. In this study, we successfully classified P-waves so that the abnormal morphologies of AF/AFL can be truly interpreted with other ECG records databases. Additionally, for the QRS complex, we achieved 100% Acc when determining the RR interval calculation. Finally, the T-wave performance results were improved compared with those from our previous works in terms of the highest Acc, Sen and Pre^18,19^. Based on these results, the possibility of other heart abnormality interpreration can be considered for early diagnosis by cardiologists.

**Table 8:** Benchmark studies for the ECG delineation performance.

Authors	Dataset	Method	Performances (%)								
			P-waves			QRS-complexes			T-waves		
			Acc	Sen	Pre	Acc	Sen	Pre	Acc	Sen	Pre
Chen et.al.^17^	LUDB	U-Net	–	99.96	–	–	100	–	–	99.68	–
Liu et al*.*^36^	LUDB	ResNet and LSTM	–	100	–	–	100	–	–	99.50	–
Moskalenko et al.^37^	LUDB	U-Net	–	98.60	–	–	99.99	–	–	99.36	–
Jimenez-Perez et al*.*^38^	LUDB	W-Net	–	99.81	99.62	–	100	100	–	100	100
Our previous work^18^	QT Database	ConvBiLSTM	99.89	98.96	99.09	99.95	99.07	99.31	99.61	98.58	98.37
Our previous work^19^	QT Database	Denoising autoencoder (DAE)-ConvBiLSTM	99.81	98.55	98.26	99.86	99.26	98.57	99.35	97.49	97.37
This work	LUDB	ConvBiLSTM with hyperparameter optimization model	100	99.97	99.97	100	99.98	99.98	99.94	99.70	99.82

Using the best model from the ECG delineation task, we were concerned with interpreting the abnormalities of morphology (AF, AFL, SBR, SVTA, T and VT). The performance results were excellent; however, there are limitations to this study. First, in all experimental studies, only single-lead ECG records (lead II) were used to generate the improved ConvBiLSTM with the GSO algorithm. We have not yet applied multilead or 12-lead ECGs for ECG delineation. Many types of heart abnormalities require a standard 12-lead ECG observation because each ECG signal has a different heart vector orientation. Second, T-wave characteristics have maximal error, which it is observed for T-wave offset, whose delineation is a well-known hard problem. Third, the generalization of the ECG delineation model using other ECG databases is extensively required, more datasets could achieve greater generalization and more robust performance.

## Conclusion

In this study, the ECG delineation task was conducted to generate an automated and robust model for ECG abnormality waveform interpretation. Using the DL approach, the performance results were improved when using the ConvBiLSTM model combined with the simplest hyperparameter tuning algorithm. The ECG waveform classification was used to classify the onset and offset P-QRS-T waves and no waves. The performance results were above 99% for Acc, Sen, and Pre. Using the grid search optimization algorithm, we simply divided the tuning domain of the hyperparameters into a discrete grid. This approach was used to determine the optimal hyperparameter that yielded the most precise prediction. From the improved ConvBiLSTM model, we experimented with a DL-based delineation model to interpret the abnormalities of the ECG waveform, i.e., AF, AFL, SBR, SVTA, T and VT. The aforementioned heart abnormalities are affected by irregular/regular heartbeats and the absence of P-waves. In all experimental datasets used in this study, the results showed that the improved ConvBiLSTM can successfully interpret AF, AFL, SBR, SVTA, T and VT infected as AF, AFL, SBR, SVTA, T and VT, respectively. The excellent results of ECG waveform classification reflect the vast opportunity to use the improved ConvBiLSTM model to analyze ECG recordings to diagnose other heart abnormalities related to ECG morphology. The existence of noise does not affect the performance of the proposed ECG delineation task. With varying morphology and features, the model is robust and can be implemented in clinical practice.

## Data Availability

The datasets generated and/or analysed during the current study are available in the PhysioNet repository (https://physionet.org/). Lobachevsky University Electrocardiography Database (LUDB) (https://physionet.org/content/ludb/1.0.1/), PhysioNet/Computing in Cardiology Challenge 2017 (https://physionet.org/content/challenge-2017/1.0.0/), China Physiological Signal Challenge 2018 (http://2018.icbeb.org/Challenge.html), ECG Arrhythmia of Chapman University (https://physionet.org/content/ecg-arrhythmia/1.0.0/) and MIT Arrhythmia Database (https://physionet.org/content/mitdb/1.0.0/). The datasets of Central General Hospital Dr. Mohammad Hoesin, Indonesia was used and/or analysed during the current study available from the corresponding author on reasonable request. All methods were carried out in accordance with relevant guidelines and regulations.

## References

[1] Wang M, Rahardja S, Fränti P, Rahardja S (2023). Single-lead ECG recordings modeling for end-to-end recognition of atrial fibrillation with dual-path RNN. Biomed. Signal Process. Control.

[2] Fleming JS (2012). Interpreting the Electrocardiogram.

[3] Liang X (2022). ECG\_SegNet: An ECG delineation model based on the encoder–decoder structure. Comput. Biol. Med..

[4] Almumri A, Balakrishnan E, Narasimman S (2021). Discrete wavelet transform based feature extraction in electrocardiogram signals. Glob. J. Pure Appl. Math..

[5] Yoon D, Lim HS, Jung K, Kim TY, Lee S (2019). Deep learning-based electrocardiogram signal noise detection and screening model. Healthc. Inform. Res..

[6] Jimenez-Perez G, Alcaine A, Camara O (2021). Delineation of the electrocardiogram with a mixed-quality-annotations dataset using convolutional neural networks. Sci. Rep..

[7] Banerjee S, Gupta R, Mitra M (2012). Delineation of ECG characteristic features using multiresolution wavelet analysis method. Measurement.

[8] Darmawahyuni A (2021). Delineation of electrocardiogram morphologies by using discrete wavelet transforms. Indones. J. Electr. Eng. Comput. Sci..

[9] Chen H, Maharatna K (2020). An automatic R and T peak detection method based on the combination of hierarchical clustering and discrete wavelet transform. IEEE J. Biomed. Health Inform..

[10] Peimankar A, Puthusserypady S (2021). DENS-ECG: A deep learning approach for ECG signal delineation. Expert Syst. Appl..

[11] Londhe AN, Atulkar M (2021). Semantic segmentation of ECG waves using hybrid channel-mix convolutional and bidirectional LSTM. Biomed. Signal Process. Control.

[12] Wang D (2023). Inter-patient ECG characteristic wave detection based on convolutional neural network combined with transformer. Biomed. Signal Process Control.

[13] Jimenez-Perez G, Alcaine A, Camara O (2010). U-Net architecture for the automatic detection and delineation of the electrocardiogram. Comput. Cardiol..

[14] Wang J, Li R, Li R, Fu B (2020). A knowledge-based deep learning method for ECG signal delineation. Future Gener. Comput. Syst..

[15] Nurmaini S (2020). Electrocardiogram signal classification for automated delineation using bidirectional long short-term memory. Inform. Med. Unlocked.

[16] Wu W, Huang Y, Wu X (2022). A new deep learning method with self-supervised learning for delineation of the electrocardiogram. Entropy.

[17] Chen Z, Wang M, Zhang M, Huang W, Gu H, Xu J (2023). Post-processing refined ECG delineation based on 1D-UNet. Biomed. Signal Process. Control.

[18] Nurmaini S (2021). Beat-to-beat electrocardiogram waveform classification based on a stacked convolutional and bidirectional long short-term memory. IEEE Access.

[19] Tutuko B (2022). DAE-ConvBiLSTM: End-to-end learning single-lead electrocardiogram signal for heart abnormalities detection. PLoS ONE.

[20] Tutuko B (2022). Short single-lead ECG signal delineation-based deep learning: Implementation in automatic atrial fibrillation identification. Sensors.

[21] Kalyakulina AI (2020). Ludb: A new open-access validation tool for electrocardiogram delineation algorithms. IEEE Access.

[22] Laguna P, Mark RG, Goldberg A, Moody GB (1997). A database for evaluation of algorithms for measurement of QT and other waveform intervals in the ECG. Comput. Cardiol..

[23] Clifford GD (2017). AF classification from a short single lead ECG recording: The PhysioNet/computing in cardiology challenge 2017. Comput. Cardiol. (CinC).

[24] Liu F (2018). An open access database for evaluating the algorithms of electrocardiogram rhythm and morphology abnormality detection. J. Med. Imaging Health Inform..

[25] Zheng J, Zhang J, Danioko S, Yao H, Guo H, Rakovski C (2020). A 12-lead electrocardiogram database for arrhythmia research covering more than 10,000 patients. Sci. Data.

[26] Zheng J (2020). Optimal multi-stage arrhythmia classification approach. Sci. Rep..

[27] Moody GB, Mark RG (2001). The impact of the MIT-BIH arrhythmia database. IEEE Eng. Med. Biol. Mag..

[28] Gragido, W., Pirc, J., Selby, N. & Molina, D. Chapter 4—Signal-to-noise ratio. In *Blackhatonomics* (Gragido, W., Pirc, J., Selby, N. & Molina, D. Eds.). 45–55 (Syngress, 2013). 10.1016/B978-1-59-749740-4.00004-6.

[29] Kiranyaz S, Avci O, Abdeljaber O, Ince T, Gabbouj M, Inman DJ (2021). 1D convolutional neural networks and applications: A survey. Mech. Syst. Signal Process.

[30] Sangaiah AK, Arumugam M, Bian G-B (2020). An intelligent learning approach for improving ECG signal classification and arrhythmia analysis. Artif. Intell. Med..

[31] Fox K (2007). Resting heart rate in cardiovascular disease. J. Am. Coll. Cardiol..

[32] Ostchega, Y., Porter, K.S., Hughes, J., Dillon, C.F. & Nwankwo, T. Resting pulse rate reference data for children, adolescents, and adults: United States, 1999–2008. In *National Health Statistics Report, No. 41* (2011). 21905522

[33] Saclova L, Nemcova A, Smisek R, Smital L, Vitek M, Ronzhina M (2022). Reliable P wave detection in pathological ECG signals. Sci. Rep..

[34] Goldberger, A.L., Goldberger, Z.D. & Shvilkin, A. *Goldberger’s Clinical Electrocardiography: A Simplified Approach: Ninth Edition* (2017).

[35] Han C, Que W, Wang S, Zhang J, Zhao J, Shi L (2022). QRS complexes and T waves localization in multi-lead ECG signals based on deep learning and electrophysiology knowledge. Expert Syst. Appl..

[36] Liu J (2022). A novel P-QRS-T wave localization method in ECG signals based on hybrid neural networks. Comput. Biol. Med..

[37] Moskalenko, V., Zolotykh, N. & Osipov, G. Deep learning for ECG segmentation. In *Advances in Neural Computation, Machine Learning, and Cognitive Research III: Selected Papers from the XXI International Conference on Neuroinformatics, October 7–11, 2019, Dolgoprudny, Moscow Region, Russia* 246–254 (2020).

[38] Jimenez-Perez, G., Acosta, J., Alcaine, A. & Camara, O. *Generalizing Electrocardiogram Delineation: Training Convolutional Neural Networks with Synthetic Data Augmentation*. arXiv preprint arXiv:2111.12996 (2021). 10.3389/fcvm.2024.1341786 PMC11294154 39100388

